# Characterizing reflux on high resolution esophageal manometry with impedance

**DOI:** 10.1186/s12876-022-02194-0

**Published:** 2022-03-08

**Authors:** Asad Jehangir, Zubair Malik, Henry P. Parkman

**Affiliations:** 1 Gastroenterology Section, Department of Medicine, Medical College of Georgia at Augusta University, Augusta, GA USA; 2 Gastroenterology Section, Department of Medicine, Temple University School of Medicine, Philadelphia, PA USA

**Keywords:** Esophageal manometry, Impedance, Reflux, GERD, Esophagogastric junction

## Abstract

**Background:**

In some patients, reflux at esophagogastric junction (EGJ) can be seen on the impedance portion of the high-resolution esophageal manometry with impedance (HREMI) studies. How this correlates with reflux on conventional esophageal reflux monitoring studies is unknown. We aimed to: (1) determine prevalence of reflux seen on HREMI, (2) correlate reflux during HREMI with reflux on esophageal reflux monitoring studies.

**Methods:**

Patients undergoing HREMI and ambulatory reflux monitoring (7/2019 to 2/2020) were studied. Healthy volunteers (HVs) underwent HREMI.

**Key results:**

Sixteen HVs underwent HREMI (no reflux on HREMI = 13, reflux on 1 swallow on HREMI = 3). Of 229 patients (mean age 56.4 ± 1.0, 68.1% females) undergoing HREMI, 47 (20.5%) had pathologic reflux at EGJ on HREMI (reflux on ≥ 2 swallows). The patients with reflux on HREMI had more frequent reflux events on multichannel intraluminal impedance-pH (MII-pH) than patients without reflux on HREMI (63.5 ± 7.1 vs 42.1 ± 2.3, *p* = 0.01). On ambulatory pH monitoring, 113 (49.3%) had GERD and 42 (18.3%) borderline results. Patients with reflux on HREMI more commonly had GERD (56.3% vs 48.6%) and borderline results (28.1% vs 18.3%) than patients without reflux on HREMI (*p* = 0.01). Reflux on ≥ 2 swallows on HREMI had a specificity of 83.6% and sensitivity of 24.8% for GERD. Reflux on ≥ 5 swallows on HREMI improved specificity to 91.4%, with sensitivity of 14.2% for GERD.

**Conclusions and inferences:**

Amongst patients undergoing HREMI, 20.5% had pathologic reflux at EGJ on HREMI. Patients with reflux on HREMI more frequently had GERD on ambulatory pH monitoring. Reflux on HREMI had good specificity but low sensitivity for GERD.

## Introduction

About a fourth of North Americans suffer from gastroesophageal reflux disease (GERD), that contributes to significant healthcare costs [1, 2]. Diagnosis of GERD is often made on clinical assessment after which an empiric trial of a proton pump inhibitor (PPI) is often prescribed [3]. In patients who fail to respond adequately to PPI or in cases of unclear diagnosis, objective testing to confirm or refute a diagnosis of GERD is needed. LA grades C and D esophagitis on esophagogastroduodenoscopy (EGD) in suggestive of GERD; but patients with reflux symptoms and normal endoscopic examination may still have non-erosive gastroesophageal reflux disease (NRED) [3]. Ambulatory reflux monitoring, with multichannel intraluminal impedance pH (MII-pH) or pH capsule, is the current gold standard for the diagnosis of NERD [4]. However, ambulatory reflux monitoring may be limited by cost, time required to performed the test, or patient tolerance [3, 5].

Esophageal pH impedance testing with MII-pH is typically preceded by esophageal manometry to help assess the location of the esophagogastric junction (EGJ) for appropriate positioning of pH impedance catheter. Some esophageal manometric parameters may also correlate with GERD. Amongst patients with non-specific motility disorders, those with GERD have lower distal contractile integral (DCI) than non-GERD patients [6]. Low lower esophageal sphincter (LES) pressures are associated with higher esophageal acid exposure time (AET) [7]. Presence of hiatal hernia on HREMI is also associated with a higher reflux burden [8]. Adding impedance measurement to esophageal manometry allows measurement of the baseline impedance, a metric associated with esophageal mucosal integrity, and detect retrograde movements (i.e. reflux) in the esophagus [9]. Patients with GERD have lower baseline impedance on high resolution esophageal manometry with impedance (HREMI) than non-GERD patients [4]. How reflux seen on the impedance portion of esophageal manometry correlates with a diagnosis of GERD is not known.

The aims of this study were to: (1) Determine the prevalence of reflux seen on HREMI, (2) Correlate reflux during HREMI studies with reflux on esophageal reflux monitoring studies.

## Materials and methods

Consecutive patients undergoing HREMI and ambulatory reflux monitoring from July 2019 to February 2020 at Temple University Hospital Motility Center for non-obstructive upper gastrointestinal (GI) symptoms, lung transplant evaluation or bariatric operation evaluation were studied. We excluded patients with prior history of esophageal operations and achalasia. Healthy volunteers (HVs) also underwent HREMI. HVs had no medical comorbidities, gastrointestinal symptoms or use of reflux medications (including PPI or H2 antagonists). The study was reviewed and approved by Temple University Hospital Institutional Review Board.

### Questionnaires on demographics and symptoms

Patients undergoing HREMI completed a questionnaire on demographics (including age, gender, height, and body weight). Patients also reported the duration of their upper gastrointestinal symptoms and rated their severity in the last 2-weeks using a modified Patient Assessment of Upper Gastrointestinal Symptoms (PAGI-SYM) questionnaire [10], on a 0 to 4 scale (0 = None, 1 = Mild, 2 = Moderate, 3 = Severe, or 4 = Very Severe). These upper gastrointestinal symptoms included dysphagia, heartburn, regurgitation, chest pain, hoarseness, coughing, belching, nausea, and vomiting. The overall symptom severity was calculated by adding severities of individual 9 symptom scores (range 0 to 36).

### High resolution esophageal manometry with impedance

Patients and HVs arrived at the endoscopy/motility unit on the morning of the study after an 8-h fast. Patients were instructed to discontinue medications 48–72 h before HREMI that could impact test results; these included prokinetics and opioids when feasible. Esophageal manometry was performed according to the standard clinical protocol at Temple University Motility Lab using a solid-state catheter consisting of 36 circumferential pressure sensors spaced 1 cm apart and 18 impedance sensors spaced 2 cm apart (ManoScan, Medtronic, Inc., Shoreview, MN, USA). The catheter was inserted via nasal intubation and advanced into the stomach with the patient sitting upright. The patient was then placed supine, positioning the catheter so that upper esophageal sphincter (UES), LES and proximal stomach were present on the computer monitor. After 5 min for catheter equilibration to body temperature, a 30 s baseline landmark recording was obtained. This was followed by 12 wet swallows with 5 cc of room temperature saline given every 30 s. Twelve swallows were performed to help ensure at least 10 swallows available for analysis. The catheter was subsequently removed, recording the pressures excorpus for subsequent thermal calibration of the catheter. The studies were systematically analyzed (ManoView software version 3.3, Medtronic, Inc.) for EGJ pressures at landmark (baseline pressures without swallowing for 30 s) along with pressure profiles during 12 wet saline swallows. The swallows were analyzed for UES resting, residual and mean peak pressures, LES resting and residual pressures, DCI, integrated relaxation pressure (IRP), and bolus clearance percentage using impedance. The HREMI tracings were reviewed to categorize the patients using the Chicago Classification (CC) version 3.0 [11]. Isobaric contour plots using a 20-mmHg pressure threshold were used to analyze the peristalsis parameters.

**Fig. 1:**
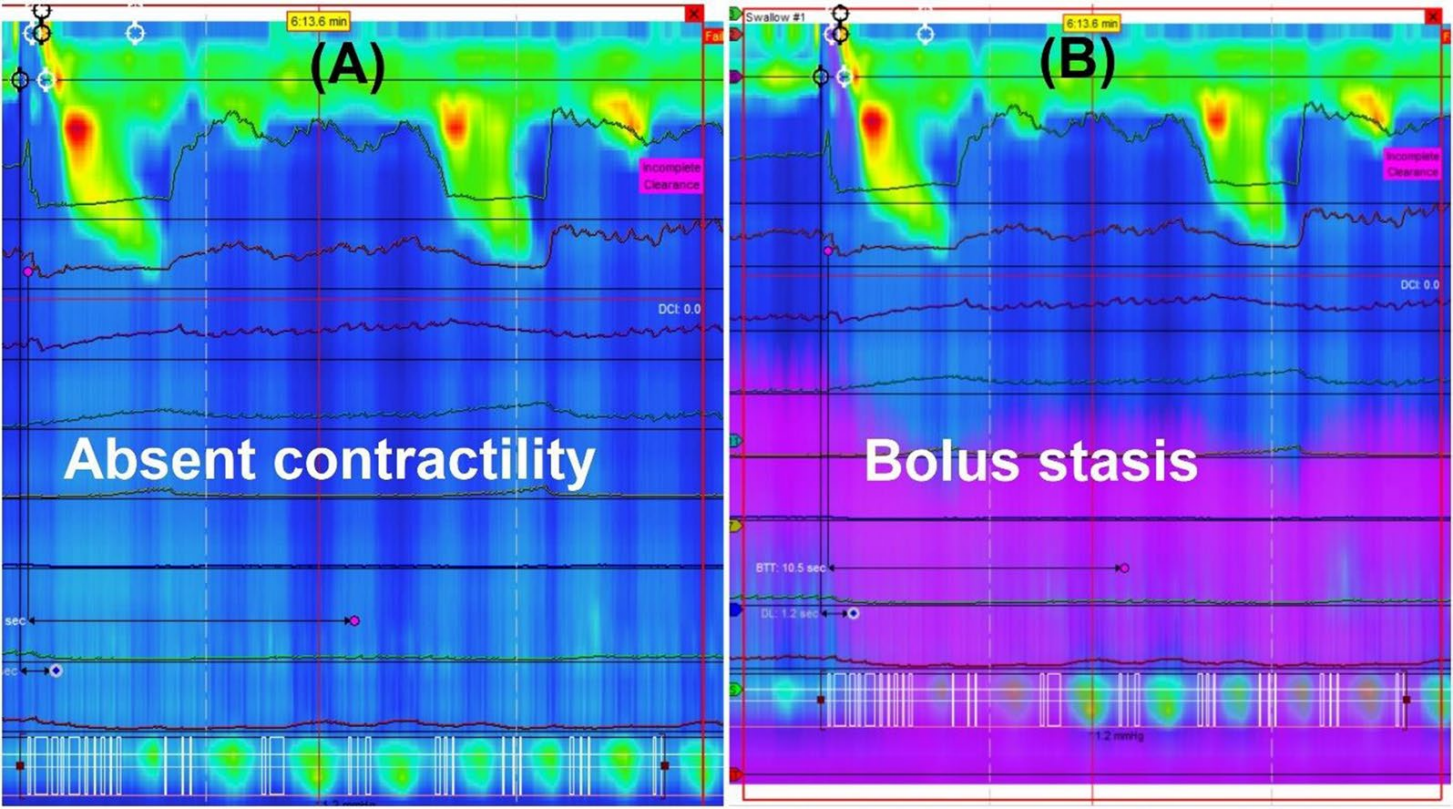
**A** High resolution manometry of a patient showing absent contractility in the mid/lower esophagus. **B** Adding impedance measurements to the same swallow showing bolus stasis

**Fig. 2:**
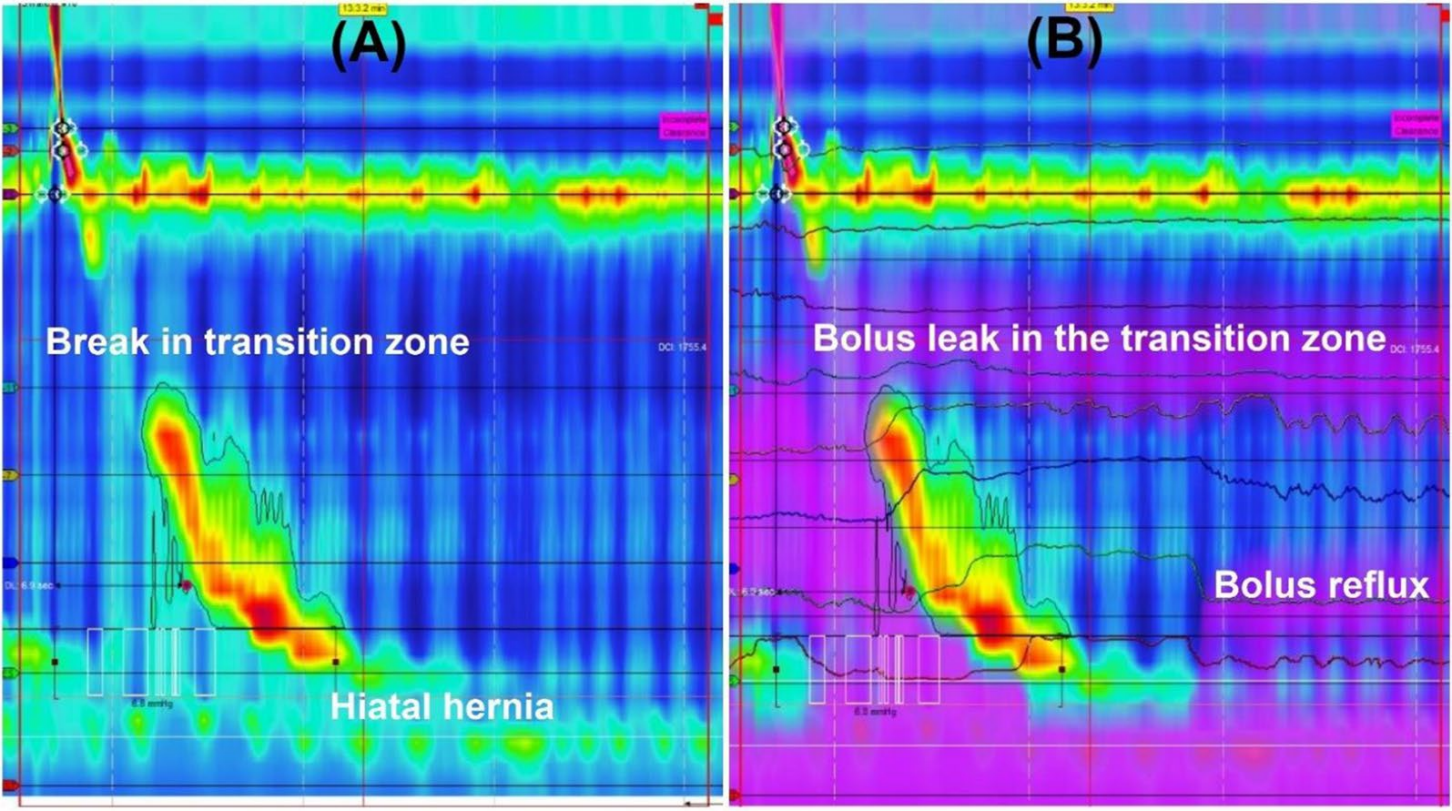
**A** High resolution manometry of a patient with reflux symptoms showing a break in the transition zone and EGJ type III morphology. **B** Adding impedance measurements to the same swallow showing some bolus retention in the transition zone as well as retrograde movement of reflux through the esophagogastric junction

**Fig. 3:**
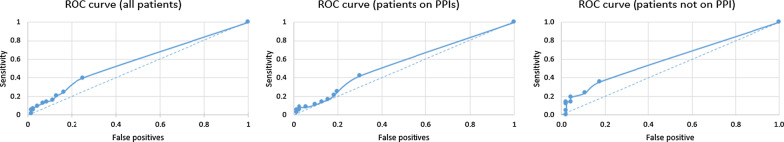
Receiver operating characteristic (ROC) analysis at different thresholds of number of swallows with reflux on high resolution esophageal manometry with impedance in predicting gastroesophageal reflux disease on ambulatory pH monitoring

The bolus (saline) flow during swallows on HREMI studies was categorized using impedance monitoring with threshold of 1.00 kOhm as: (1) Normal: if ≥ 80% (≥ 10) swallows had complete bolus clearance, (2) Bolus stasis: if there was bolus retention in the esophageal body in > 20% (≥ 3) swallows as characterized by persistent purple shade and lack of return of impedance to baseline after a swallow (Fig. 1), (3) Bolus retention at transition zone: if there was bolus retention at the transition between striated and smooth muscles in the esophagus, and/or (4) Reflux at EGJ (if there was retrograde movement of bolus from the stomach into the esophagus as characterized by return of impedance to baseline after a swallow followed by a ≥ 50% decrease in impedance as detected by ≥ 2 impedance sensors located closest to the proximal border of the LES within 30 s i.e. before the subsequent swallow (Fig. 2). If esophageal shortening was seen post-swallow, reflux on HREMI was defined as retrograde movement of bolus detected in 2 impedance sensors above the proximal LES border of the shortened esophagus. Esophageal shortening was defined as elevation of the lower esophageal sphincter ≥ 3 cm as previously reported by Biasutto et al. [12], as up to 3 cm of proximal LES migration has been reported with swallows in healthy patients [13]. All manometric studies were initially reviewed by author A.J., and then reanalyzed by author Z.M. or H.P.P.

### Ambulatory reflux monitoring

The decision to perform ambulatory reflux monitoring after HREMI using MII-pH or Bravo™ Reflux Capsule was made by the physician assessing the patients, on or off PPI, as clinically indicated. The patients who underwent ambulatory reflux monitoring off PPIs were asked to hold PPIs for 2 weeks before the test. Ambulatory reflux monitoring was not performed in HVs as we used GERD criteria defined by the international consensus [3]. In patients undergoing MII-pH, an esophageal pH probe (Medtronic Inc., Shoreview, Minneapolis, MN, USA) was placed after topical nasal anesthesia, so that the distal pH probe was positioned 5 cm above the proximal portion of the lower esophageal sphincter. The probe was attached to an external electronic data recorder (Digitrapper™ pH-Z, Medtronic Inc., Shoreview, Minneapolis, MN, USA) for continuous 24-h esophageal pH monitoring. The probe was removed the following day, and pH data were downloaded for analysis (AccuView™ Reflux Software v6.1, Medtronic, Inc., Shoreview, Minneapolis, MN, USA). Patients undergoing MII-pH testing were diagnosed with GERD using Lyon consensus, i.e. esophageal acid exposure time > 6.0% and/or > 80 reflux episodes detected by impedance [3].

In some patients, ambulatory reflux monitoring was performed using Bravo™ Reflux Capsule placed on EGD 6 cm above the squamocolumnar line. The patients returned Bravo™ Reflux Recorder after 48–96 h of reflux monitoring, and the data was downloaded using Reflux Software v6.1. Patients undergoing Bravo™ reflux monitoring were instructed to stop PPIs for 2 weeks prior to and during the procedure, and diagnosis of GERD was made if the AET was > 6%.

### Data management and statistical analysis

A retrospective review of the questionnaires and HREMI was performed after compiling these in Microsoft Excel database. Mann Whitney *U* test was used to compare symptoms recorded on ordinal scale and for quantitative data. These results are expressed as mean ± standard error of mean. Chi-squared test was used for categorical data, with results expressed as percentages. Unanswered questions were excluded from the analyses. p values < 0.05 were considered statistically significant. No adjustment was made for multiple comparisons in this exploratory study.

## Results

### Demographics and upper gastrointestinal symptoms

Of a total of 247 patients undergoing HREMI and esophageal reflux monitoring studies [14], thirteen with achalasia (Type 1 Achalasia = 3, Type II Achalasia = 3, Type III Achalasia = 7) and five with history of esophageal operations (Heller myotomy = 2, Peroral Endoscopic Myotomy = 2, Heller myotomy with fundoplication = 1) were excluded. In Table 1, we performed analyses of the remaining 229 patients included in this study with upper GI symptoms who had HREMI and esophageal reflux monitoring studies performed. Patients with reflux on HREMI had a higher mean BMI (33.3 ± 1.5 vs 27.4 ± 0.4, *p* = 0.001) than patients without reflux on HREMI. The most severe upper GI symptoms in patients undergoing HREMI included heartburn (1.4 ± 0.1), coughing (1.4 ± 0.1), regurgitation (1.1 ± 0.1), nausea (1.1 ± 0.1), dysphagia (0.9 ± 0.1), chest pain (0.8 ± 0.1) and vomiting (0.7 ± 0.1). On comparison of upper GI symptoms, patients without reflux on HREMI had higher severity of regurgitation (1.2 ± 0.1 vs 0.8 ± 0.1, *p* = 0.033) compared to patients with reflux on HREMI. The overall duration of upper GI symptoms in patients undergoing HREMI was 7.1 ± 0.7 years with no significant differences between patients with or without reflux on HREMI.

**Table 1:** Demographics and symptom severities of patients undergoing high resolution esophageal manometry with impedance testing and ambulatory pH reflux monitoring for upper GI symptoms, including all patients and patients stratified by presence or absence of pathologic reflux on HREMI

	All patients (n = 229)	Patients with reflux on HREMI (n = 47)	Patients without reflux on HREMI (n = 182)	*p* value
*Demographics*				
Age	56.4 ± 1.0	57.5 ± 1.5	56.1 ± 0.9	0.770
Gender (females)	156 (68.1%)	35 (74.5%)	121 (66.5%)	0.295
BMI (kg/m^2^)	28.7 ± 0.5	33.3 ± 1.5	27.4 ± 0.4	**0.001**
*Upper Gastrointestinal Symptom*				
Heartburn	1.4 ± 0.1	1.4 ± 0.2	1.4 ± 0.1	0.809
Chest Pain	0.8 ± 0.1	0.8 ± 0.2	0.9 ± 0.1	0.845
Dysphagia	0.9 ± 0.1	0.8 ± 0.1	1.0 ± 0.1	0.434
Regurgitation	1.1 ± 0.1	0.8 ± 0.1	1.2 ± 0.1	**0.033**
Hoarseness	0.7 ± 0.1	0.5 ± 0.1	0.7 ± 0.1	0.087
Coughing	1.4 ± 0.1	1.3 ± 0.2	1.4 ± 0.1	0.699
Belching	0.9 ± 0.1	0.7 ± 0.1	1.0 ± 0.1	0.181
Nausea	1.1 ± 0.1	0.9 ± 0.1	1.1 ± 0.1	0.671
Vomiting	0.7 ± 0.1	0.4 ± 0.1	0.8 ± 0.1	0.169
Overall symptom severity^†^	8.1 ± 0.4	6.9 ± 0.8	8.5 ± 0.4	0.139
Duration of symptoms (years)	7.1 ± 0.7	6.0 ± 1.1	7.4 ± 8.5	0.442

Results expressed as % (*n*) or mean ± standard error of mean as appropriate; p value (comparing patients with reflux on HREMI to patients without reflux on HREMI) calculated using Mann Whitney U test, Student’s t test or Chi Squared test as appropriate. ^†^Overall symptom severity calculated by adding severities of individual 9 symptom scores (range 0 to 36). Abbreviations: BMI (Body Mass Index), HREMI (High Resolution Esophageal Manometry with Impedance)

### High resolution esophageal manometry with impedance

Of sixteen HVs (mean age 32.8 ± 2.6 years, 75% males) undergoing HREMI, 13 (81.3%) HVs did not have reflux on HREMI and 3 (18.8%) HVs had reflux on 1 swallow each on HREMI. As none of the HVs had reflux on more than 1 swallow, we defined reflux on ≥ 2 swallows as pathologic reflux in patients undergoing HREMI.

Amongst 229 patients undergoing HREMI and included in the study, 28 had disorders of EJG Outflow Obstruction, 41 had major disorders of peristalsis (Absent Contractility = 27, Jackhammer Esophagus = 10, Distal Esophageal Spasm = 4), 51 had minor disorders of peristalsis (Ineffective Esophageal Motility = 45, Fragmented Peristalsis = 6), while 109 had no abnormalities per CC. Patients without reflux on HREMI were more likely to have EGJOO (20.5% vs 2.1%) and major disorders of peristalsis (19.5% vs 6.4%) compared to patients with reflux on HREMI who were more likely to have minor disorders of peristalsis or no abnormalities per CC (91.5% vs 60%, *p* < 0.01, Table 2).

**Table 2:** Manometric findings of patients undergoing high resolution esophageal manometry with impedance testing, including all patients and patients stratified by presence or absence of pathologic reflux on HREMI (table excludes patients who did not undergo ambulatory pH reflux monitoring, did not have upper GI symptoms or did not complete questionnaire on GI symptoms)

Manometric Findings	All patients (n = 229)	Patients with reflux on HREMI (n = 47)	Patients without reflux on HREMI (n = 182)	*p* value
UES basal pressure (mmHg) (nl. 34–104 mmHg)	75.4 ± 2.7	80.8 ± 7.6	74.0 ± 5.5	0.843
UES residual pressure (mmHg) (< 12 mmHg)	3.0 ± 0.3	4.6 ± 0.7	2.6 ± 0.2	**0.029**
UES mean peak pressure (mmHg) (nl. < 19.5 mmHg)	12.9 ± 0.6	13.0 ± 1.3	12.8 ± 1.0	0.601
DCI (nl. 450–8000 mmHg^.^s^.^cm)	1632.2 ± 112.6	1396.0 ± 231.3	1677.7 ± 124.4	0.460
LES basal pressure (nl. 13–43 mmHg)	26.5 ± 1.0	16.3 ± 1.9	29.2 ± 2.2	**< 0.001**
IRP (nl. < 15 mmHg)	10.1 ± 0.5	6.4 ± 1.1	11.0 ± 0.8	**0.001**
EGJ type II/III morphology	111 (48.5%)	36 (76.6%)	75 (41.2%)	**< 0.001**
Bolus clearance (normal > 80%)	60.7 ± 2.4%	69.0 ± 4.4	58.5 ± 4.3	0.353
Chicago classification				
EGJOO	28 (12.2%)	1 (2.1%)	27 (20.5%)	**0.001**
Major Disorders	41 (17.9%)	3 (6.4%)	38 (19.5%)	
Minor Disorders/no abnormalities	160 (87.8%)	43 (91.5%)	117 (60%)	

Results expressed as % (*n*) or mean ± standard error of mean as appropriate; p value (comparing patients with reflux on HREMI to patients without reflux on HREMI) calculated using Mann Whitney U test, Student’s t test or Chi Squared test as appropriate. Abbreviations: DCI (Distal Contractile Integral), EGJOO (Esophagogastric Junction Outflow Obstruction), HREMI (High Resolution Esophageal Manometry with Impedance), IRP (Integrated Relaxation Pressure), LES (Lower Esophageal Sphincter)

The average number of swallows performed amongst all patients were 11.9 ± 0.1 per patient. On assessment of bolus flow using impedance measurements, 61 patients (25.2%) had normal bolus flow on HREMI, while 168 patients (74.8%) had abnormal bolus transit on impedance analysis including 95 (41.5%) patients with bolus stasis in the esophagus on ≥ 3 swallows, 64 (27.9%) patients with bolus retention at the transition zone and/or 47 (20.5%) patients with pathologic bolus reflux at EGJ. Amongst 47 patients with pathologic reflux on HREMI, 29 patients had bolus reflux only, 7 patients had bolus reflux and retention at transition zone, 10 patients had bolus reflux and stasis, while 1 patient had bolus reflux, stasis and retention at transition zone. The patients with reflux on HREMI had on average reflux seen on 4.0 ± 0.3 swallows (reflux on 1 swallow = 27 patients, 2 swallows = 9 patients, 3 swallows = 7 patients, 4 swallows = 5 patients, 5 swallows = 4 patients, 6 swallows = 6 patients, 7 swallows = 5 patients, 8 swallows = 1 patient, 9 swallows = 1 patient, 10 swallows = 1 patient, 11 swallows = 4, 12 swallows = 4 patients). Patients with pathologic reflux on HREMI had lower esophageal sphincter (LES) basal pressure (16.3 ± 1.9 vs. 29.2 ± 2.2 mmHg), integrated relaxation pressure/IRP (6.4 ± 1.1 mmHg vs 11.0 ± 0.8 mmHg), and more likely to have EGJ type II or III morphology (76.6% vs 41.2%) than patients without reflux on HREMI (all *p* ≤ 0.01, Table 2). Patients with reflux on HREMI had higher UES residual pressure (4.6 ± 0.7) than patients without reflux on HREMI (2.6 ± 0.2, *p* = 0.03). There were no significant differences in the UES resting and mean peak pressures between patients with and without reflux on HREMI.

### Ambulatory reflux monitoring

Of the 229 patients with upper GI symptoms and ambulatory pH monitoring (MII-pH = 219, Bravo = 10), 113 (49.3%) had GERD per Lyon consensus criteria (Table 3). Pathologic reflux on HREMI was seen in about a fourth (*n* = 28, 24.8%) of patients with GERD per Lyon criteria. Patients with reflux on HREMI were significantly more likely to meet Lyon consensus criteria for GERD (56.3% vs 48.6%) and have borderline results (28.1% vs 18.3%) on ambulatory reflux monitoring than patients without reflux on HREMI *p* = 0.01). Patients with reflux on HREMI had a higher number of reflux events detected on MII-pH than patients without reflux on HREMI (63.5 ± 7.1 vs 42.1 ± 2.3, *p* = 0.01). Patients with reflux on HREMI trended to have a higher esophageal acid exposure time on esophageal pH impedance testing than patients without reflux on HREMI (11.6 ± 1.8% vs 9.4 ± 1.0% respectively, *p* = 0.07).

**Table 3:** Ambulatory pH reflux monitoring results of patients undergoing HREMI and MII-pH or Bravo testing, and stratified by presence or absence of reflux on high resolution esophageal manometry with impedance testing (all patients, and patients on and off PPI)

Ambulatory esophageal reflux monitoring	All patients (n = 229)	Patients with reflux on HREMI (n = 47)	Patients without reflux on HREMI (n = 182)	*p* value
Esophageal AET	9.9 ± 0.9%	11.6 ± 1.8%	9.4 ± 1.0%	0.074
Reflux episodes on MII-pH	46.7 ± 2.3	63.5 ± 7.1	42.1 ± 2.3	**0.006**
Lyon classification				
GERD	113 (49.3%)	28 (59.6%)	85 (46.7%)	**0.014**
Borderline	42 (18.3%)	12 (25.5%)	30 (16.5%)	
Normal	74 (32.3%)	7 (14.9%)	67 (26.8%)	

	Patients on PPI (n = 141)	Patients with reflux on HREMI (n = 32)	Patients without reflux on HREMI (n = 109)	*p* value
Esophageal AET	10.1 ± 1.1%	10.9 ± 2.2%	9.8 ± 1.3%	0.298
Reflux episodes on MII-pH	48.0 ± 3.3	61.8 ± 9.5	44.0 ± 3.1	0.190
Lyon classification				
-GERD (AET)	71 (50.4%)	18 (56.3%)	53 (48.6%)	0.135
-Borderline	29 (20.6%)	9 (28.1%)	20 (18.3%)	
-Normal	41 (29.1%)	5 (15.6%)	36 (33%)	

	Patients off PPI (n = 88)	Patients with reflux on HREMI (n = 15)	Patients without reflux on HREMI (n = 73)	*p* value
Esophageal AET	9.5 ± 1.3%	13.1 ± 3.4%	8.8 ± 1.4%	0.126
Reflux episodes on MII-pH	44.3 ± 3.5	67.1 ± 9.8	38.8 ± 3.4	**0.007**
Lyon classification				
GERD	42 (47.7%)	10 (66.7%)	32 (42.5%)	0.105
Borderline	13 (14.8%)	3 (20%)	10 (13.7%)	
Normal	33 (37.5%)	2 (13.3%)	31 (42.5%)	

Results expressed as *n* (%) or mean ± standard error of mean as appropriate; p value (comparing patients with reflux on HREMI to patients without reflux on HREMI) calculated using Student’s t test or Chi Squared test as appropriate. Abbreviations: AET (Acid Exposure Time), GERD (Gastroesophageal Reflux Disease), HREMI (High Resolution Esophageal Manometry with Impedance), MII (Multichannel intraluminal impedance), PPI (Proton Pump Inhibitor).

Over half (*n* = 141, 61.5%) of the patients undergoing esophageal pH impedance monitoring were taking PPI at the time the test was performed. On further analysis of 141 patients undergoing esophageal reflux monitoring on PPI, patients with reflux on HREMI had quantitatively higher number of reflux events on MII-pH than patients without reflux on HREMI (61.8 ± 9.5 vs 44.0 ± 3.1, *p* = 0.19). The patients with reflux on HREMI had a trend to more commonly meet Lyon consensus GERD criteria (56.3% vs 48.8%) and have borderline results (28.1% vs 18.3%) compared to patients without reflux on HREMI (*p* = 0.135). On analysis of 88 patients off PPI during esophageal reflux monitoring, patients with reflux on HREMI had significantly higher number of reflux events on MII-pH than patients without reflux on HREMI (67.1 ± 9.8 vs 38.8 ± 3.4, *p* = 0.01). The patients with reflux on HREMI trended to be more likely to meet GERD criteria per Lyon consensus (66.7% vs 42.5%) and borderline results (20% vs 13.7%) than patients without reflux on HREMI (*p* = 0.11).

The presence of a type II or III EGJ morphology on HREMI was associated with higher AET (12.0 ± 1.3% vs 7.9 ± 1.0%, *p* = 0.02), higher number of reflux events on MII-pH testing (55.5 ± 3.4 vs 38.0 ± 3.2, *p* < 0.01), and more frequent diagnosis of GERD per Lyon consensus (58.6% vs 40.7%, *p* = 0.01) compared to patients with type I EGJ morphology (Table 4).

**Table 4:** Reflux on high resolution esophageal manometry with impedance testing and ambulatory pH monitoring with impedance testing: patients stratified by type of EGJ morphology

	EGJ type I morphology (*n* = 118)	EGJ type II/III morphology (*n* = 111)	*p* value
Reflux on HREMI	11 (9.3%)	36 (32.4%)	**< 0.001**
Esophageal Acid Exposure Time	7.9 ± 1.0	12.0 ± 1.3	**0.007**
Reflux events detected on MII- pH	38.0 ± 3.3	55.5 ± 3.4	**< 0.001**
Lyon classification			
GERD	48 (40.7%)	65 (58.6%)	**0.006**
Borderline	21 (17.8%)	21 (18.9%)	
Normal	49 (41.5%)	25 (22.5%)	

Results expressed as *n* (%) or mean ± standard error of mean as appropriate; p value (comparing patients with type I EGJ morphology with type II and III EGJ morphologies) calculated using Student’s t test or Chi Squared test as appropriate. Abbreviations: GERD (Gastroesophageal Reflux Disease), HREMI (High Resolution Esophageal Manometry with Impedance), MII (Multichannel intraluminal impedance)

Pathologic reflux on HREMI (i.e. reflux on ≥ 2 swallows) had a specificity of 83.6% and sensitivity of 24.8% for GERD (Table 5). Reflux on ≥ 5 swallows on HREMI improved specificity to 91.4%, with sensitivity of 14.2% for GERD. Reflux on all 12 swallows had specificity of 98.3% but sensitivity of 1.8% for GERD. On sub-analysis of patients on PPI at the time of esophageal reflux monitoring, pathologic reflux on HREMI had a fair specificity (80.0%) and low sensitivity (25.4%) for GERD. In patients off PPI at the time of esophageal reflux monitoring, pathologic reflux on HREMI had specificity of 89.1% and sensitivity of 23.8% for GERD. Given the low sensitivity of reflux on HREMI to diagnose GERD, the receiver operating characteristic (ROC) curves were expectedly close to the 45-degress diagonal of ROC space despite good specificity of HREMI for GERD. This was true for all patients, patients on and off PPI (Fig. 3).

**Table 5:** Sensitivity and specificity of reflux seen on high resolution esophageal manometry with impedance testing for gastroesophageal reflux disease as diagnosed by ambulatory pH monitoring with impedance testing or Bravo capsule

# of swallows on HREMI with reflux	All patients (*n* = 229)		Patients on PPI (*n* = 141)		Patients off PPI (*n* = 88)	
	Sensitivity (%)	Specificity (%)	Sensitivity (%)	Specificity (%)	Sensitivity (%)	Specificity (%)
1	39.8	75.0	42.3	70.0	35.7	82.6
2	24.8	83.6	25.4	80.0	23.8	89.1
3	20.4	87.1	21.1	81.4	19.0	95.7
4	15.9	88.8	16.9	84.3	14.3	95.7
5	14.2	91.4	14.1	87.1	14.3	95.7
6	12.4	93.1	11.3	90.0	14.3	97.8
7	9.7	95.7	8.5	94.3	11.9	97.8
8	7.1	97.4	8.5	97.1	11.9	97.8
9	6.2	97.4	7.0	97.1	11.9	97.8
10	5.3	97.4	5.6	97.1	11.9	97.8
11	5.3	98.3	5.6	98.6	4.8	97.8
12	1.8	98.3	2.8	98.6	0	97.8

*AET* Acid Exposure Time, *BID* twice daily, *HREMI* High Resolution Esophageal Manometry with Impedance, *PPI* Proton Pump Inhibitor, *QD* once daily

## Discussion

Patients with symptoms of gastroesophageal reflux disease may require esophageal function testing with esophageal manometry and ambulatory reflux monitoring. Reflux can be seen at times on impedance during HREMI studies. How reflux seen on HREMI correlate with ambulatory reflux monitoring is not known and was the subject of this study. Amongst patients undergoing HREMI, about a fifth (20.5%) had reflux at EGJ seen on HREMI. These patients had lower LES pressures and more frequent EGJ type II or III morphology on HREMI than patients without reflux on HREMI. Patients with reflux on HREMI were more likely to meet criteria for GERD on ambulatory pH monitoring. Reflux on HREMI had good specificity but low sensitivity for GERD.

The patients with reflux on HREMI had a higher BMI than patients without reflux on HREMI. Other studies have suggested a higher prevalence of GERD symptoms with increasing BMI [15–17]. The patients with reflux on HREMI had lower severity of regurgitation compared to patients without reflux on HREMI. The lower severity of regurgitation in patients with reflux on HREMI may be due to lower prevalence of EJGOO and major disorder of peristalsis compared to patients without reflux on HREMI.

We found low LES pressures in patients with reflux on HREMI compared to patients without GERD. In a study by Rengarajan et al., low LES pressures was associated with higher reflux burden [7]. In our study, patients with reflux on HREMI were more likely to have EGJ type II or III morphology (76.6%) than patients without reflux on HREMI (41.2%, *p* < 0.01). Presence of EGJ type II or III morphology on HREMI was associated with longer esophageal AET, higher number of reflux episodes on MII-pH testing, and more frequent diagnosis of GERD. Tolone et al. similarly found that increasing separation between LES and crural diaphragm was associated with higher AET, more frequent reflux episodes and diagnosis of GERD [8].

The patients with reflux on HREMI were more likely to have GERD on esophageal pH impedance testing (59.6% vs 46.7%) and borderline results (25.5% vs 16.5%), with higher number of 24-h reflux episodes on impedance testing (63.5 ± 7.1 vs 42.1 ± 2.3) compared to patients without reflux on HREMI (*p* ≤ 0.01). The AET in patients with reflux on HREMI trended to be higher in than patients without reflux on HREMI but did not reach statistical significance (11.6 ± 1.8% vs 9.4 ± 1.0%, *p* = 0.07). The patients without reflux were more likely to have major disorders of peristalsis that may have predisposed these patients to esophageal stasis and fermentation.

Our study shows that reflux seen on HREMI has good specificity though low sensitivity for GERD. Hence, while the absence of reflux on HREMI does not rule out GERD, pathologic reflux seen on HREMI may be supportive of GERD in some patients. In clinical practice, some patients poorly tolerate the MII-pH catheter, resulting in reflux monitoring data of limited duration which may be difficult to interpret. These patients often undergo Bravo testing, which requires endoscopic placement and may be associated with post procedural chest discomfort. In some of these patients, who have reflux on ≥ 5 swallows on HREMI, a presumptive diagnosis of GERD can be made with good specificity.

Our study has some limitations. This study includes a heterogeneous group of patients with non-obstructing upper GI symptoms, those undergoing evaluations for lung transplant and bariatric operations. The HVs in our study were younger and less likely to be females than the patients undergoing HREMI. Our criteria for pathologic reflux on HREMI need further validation in age- and gender-matched controls. The HVs in our study did not undergo esophageal reflux monitoring and we used the international consensus to define GERD [3]. While all patients previously had history of undergoing upper, but we did not include endoscopic findings of these patients in our study. Over a third (41.5%) of the patients had abnormal bolus stasis on HREMI, and it is possible that bolus stasis may have impaired assessment of reflux on swallows on HREMI in some patients. Symptom index or symptom association probabilities were not assessed in this study. Baseline impedance was not measured in the study to see if it correlates with the reflux episodes seen on HREMI. Reflux episodes on HREMI do not necessarily equate to reflux observed with transient lower esophageal sphincter relaxations, a frequent driver of reflux events in patients with GERD [18]. Patients with reflux on HREMI were significantly more likely to have EGJ type II and III morphology on HREMI, and it is plausible that the reflux observed in some of these patients on HREMI represents reflux from the hiatal hernia. We did not measure the proximal extent of reflux observed on HREMI to see if patients with reflux reaching the proximal esophagus on HREMI had more severe GERD symptoms or longer acid esophageal exposure times on esophageal reflux monitoring. Over half (61.6%) of the patients undergoing ambulatory pH monitoring were on PPI at the time the procedure was performed. Nonetheless, patients off PPI had higher number of reflux episodes on multichannel intraluminal impedance and had a trend to more frequently meet Lyon consensus criteria for GERD. In addition, reflux on HREMI had good specificity for GERD irrespective of whether patients were on and off PPI at the time of esophageal reflux monitoring. Instead of the common GERD self-reported questionnaires such as gastroesophageal reflux disease questionnaire (GERDQ), Reflux Disease Questionnaire (RDQ) and Reflux Severity Index (RSI) questionnaires, patients reported the severities of their upper GI symptoms using a modified PAGI-SYM questionnaire. This questionnaire has not been validated but has been previously used to assess upper GI symptoms in patients with esophageal dysmotility and reflux disease [19, 20]. Lastly, the HREMI findings were interpreted using CC Version 3.0, as this study was performed before the recent Chicago Classification Version 4.0 was published and it is plausible that some of the patients classified as having ineffective esophageal motility had normal manometry per CC 4.0 [21].

In conclusion, amongst patients undergoing HREMI, about a fourth have pathologic (≥ 2 swallows) reflux at EGJ on HREMI. Patients with reflux on HREMI are more likely to meet criteria for GERD on ambulatory pH monitoring. Reflux on HREMI had good specificity for GERD, although low sensitivity. Reflux seen on HREMI may be supportive of GERD in some patients.

## Data Availability

The datasets used and/or analyzed during the current study are available from the corresponding author on reasonable request.

## Abbreviations

CC: Chicago Classification
DCI: Distal contractile integral
AET: Esophageal acid exposure time
EGD: Esophagogastroduodenoscopy
EGJ: Esophagogastric junction
GERD: Gastroesophageal reflux disease
HVs: Healthy volunteers
HREMI: High resolution esophageal manometry with impedance
IRP: Integrated relaxation pressure
LES: Lower esophageal sphincter
MII-pH: Multichannel intraluminal impedance pH
NRED: Non-erosive gastroesophageal reflux disease
PAGI-SYM: Patient Assessment of Upper Gastrointestinal Symptoms
PPI: Proton Pump inhibitor
UES: Upper esophageal sphincter
